# Digital Image Correlation of Tensile Properties for Monel 400/SS 316L Dissimilar Metal Welding Joints

**DOI:** 10.3390/ma14061560

**Published:** 2021-03-22

**Authors:** Cherish Mani, Sozharajan Balasubramani, Ramanujam Karthikeyan, Sathish Kannan

**Affiliations:** 1 Department of Mechanical Engineering 1, BITS Pilani, Dubai Campus, P.O. Box 345055 Dubai, United Arab Emirates; Sozharajanram@gmail.com; 2 Department of Mechanical Engineering, American University of Sharjah, P.O. Box 26666 Sharjah, United Arab Emirates; skannan@aus.edu

**Keywords:** DIC, tensile properties, dissimilar metal welding, Monel alloy 400, SS 316L, SEM, dimple density

## Abstract

Dissimilar metal weld joints of Monel 400 and Stainless Steel 316L stainless steel were carried out using Gas Tungsten Arc Welding (GTAW). Conventional annealing and cryogenic treatment were performed on the welded joints. Weld joints of this combination of materials have enormous potential applications in power industry and the available related literature is limited. In the present study, the tensile properties of heat treated (HT), cryotreated (CT), and untreated (UT) specimens were studied. The engineering stress and strain were determined experimentally as per Standard Test Methods for Tension Testing of Metallic Materials (ASTM E8). The strain distribution was evaluated at different zones of weld joint was evaluated using Digital Image Correlation (DIC). Significant difference was noticed between the zones. Weld zone of all samples had less local stress and strain and SS 316L heat affected zone (HAZ) zone had more local stress and strain when compared to other zones. The local strain distribution along distance from weld center line and local stress-strain curves of different zones are also predicted. Scanning Electron Microscopy was used to analyze the fracture behavior of welded samples for HT, CT, and UT specimens.

## 1. Introduction

Dissimilar metal weld joints between austenitic stainless steels and nickel alloys are extensively utilized in many medium to high temperature applications in power, energy conversion systems, and oil and gas industries [1]. Gas Tungsten Arc Welding (GTAW) is a better option for joining of austenitic stainless steel based weld joints. The selection of voltage and current plays an important role in gas tungsten arc welding of dissimilar metal welds. Higher voltage may lead to burn-through in thin sheets and lower voltage may lead to lack of fusion. Higher welding current increases heat input in the welding pool, causing increase in depth and width of weld [2]. Mohammed et al. [3] analyzed austenitic and duplex stainless steel-based welded joints using a GTAW process. The heat input required is different for the two materials considered for the study. Based on the experiments it was observed that, lower heat input joints have shown better tensile properties. Gaffar et al. [4] studied the characterization of welded joints of dissimilar steels. They have used microstructural analysis on the weld zone to study the quality of the joint. It was stated that higher strength and superior joint quality were achieved with the process of GTAW welding using the stainless steel as filler metal. Perez et al. [5] noticed that the satisfactory dissimilar welding of mild steel with stainless steels has been achieved with GTAW and metal inert gas (MIG) welding processes and better tensile strength was achieved with GTAW process. Post weld heat treatment is considered as a method for strengthening materials and it can be used to improve some of the mechanical properties, such as enhancement of strength, hardness, wear resistance, machinability, and formability [6]. Tensile properties and fracture behavior of stainless steels are determined by the relevant microstructure which is a function of the chemical composition and the applied heat treatment. The effect of heat treatment on AISI 304 stainless steel joints was investigated by Nasir et al. [7]. The tensile strength has been improved by grain size reduction and improved grain boundaries. Singh et al. [8] analyzed the effect of cryogenic treatment on Ti-6Al-4V alloy and stated that the hardness was increased by 2.5 HRC and there is a slight decrease in the compressive strength after cryogenic treatment. Vengatesh et al. [9] reviewed cryogenic treatment on steels and has reported increased thermal conductivity, hardness and wear resistance, along with reduced residual stress. Homogeneous crystal structure and precipitation of ultrafine carbides were observed after cryogenic treatment of steels. The cryogenic treatment has the capability to improve the mechanical properties of metals, but unfortunately it has resulted in reduced resistance to the corrosion. Deep cryogenic treatment has dramatically improved the wear resistance at high temperature.

Peng-Yan Sun et al. [10] used digital image correlation (DIC) method for Ti based welded joints. The strain distribution was found to be heterogenous and the deformation on both sides of the weld centerline was found to be symmetrical. Although, few studies were already reported on austenitic stainless steel with nickel alloys, studies on Monel 400 alloy and SS 316L stainless steel are very limited and the analysis of post weld treatments such as heat treatment and cryogenic treatment on tensile behavior of Monel 400 and SS 316L has not been studied so far. Since these dissimilar weld joints of SS 316L and Monel 400 weld have wider applications in petrochemical, oil, and gas sectors, it has been decided to study the effect of post weld treatments by gas tungsten arc welding. Authors in their previous study arrived suitable welding conditions and bevel geometry for the said combination [11]. Those conditions were used in the present work for dissimilar metal welding. In the present study, Monel 400 alloy and SS 316L weld joints were subjected to post weld treatment and cryogenic treatment and their effect on tensile properties were analyzed using Digital Image Correlation (DIC) and fractography along with dimple density analysis.

## 2. Materials and Methods

**Figure 1:**
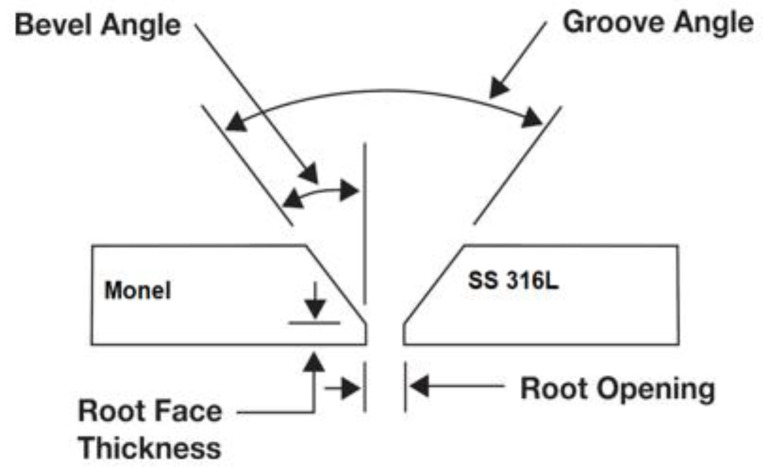
Weld groove typical section.

**Figure 2:**
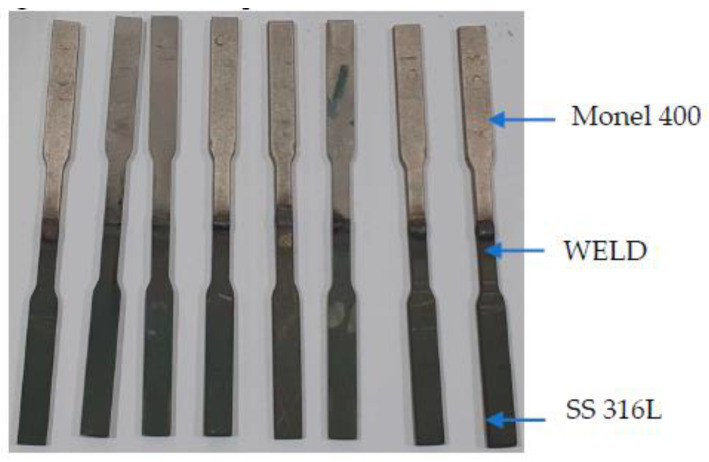
Tensile test specimens.

Weld plates of size 120 mm (Length) × 200 mm (Width) × 3.2 mm (breath) for Monel 400 and SS 316L (Nexus, Bombay, India) were welded using GTAW process using ERNiCrFe-5 (Weldwire, Pennsylvania, PA, USA) at 14 V DC with Lincoln TIG 275 welding machine (Lincoln Electric Company, Cleveland, OH, USA). Table 1 and Table 2 show the chemical composition and material properties of the materials under consideration. ERNiCrFe-5 is a nickel based electrode which is widely used for welding of nickel-chromium-iron alloys to themselves and in dissimilar welding between Ni–Cr alloys and austenitic stainless steels. Argon (Brother gas, Dubai, UAE) has been used as shielding gas, as well as backing gas. Figure 1 and Table 3 show the welding parameters and weld bevel geometry employed for the process. Nondestructive testing by radiography techniques (Inspec, Dubai, UAE) was carried out on the welded plate to ensure defect free region for specimen preparation. The specimens for tensile testing have been cut out from the regions from the defect free regions using waterjet cutting (Zayani Waterjet, Dubai, UAE) as shown in Figure 2.

## 3. Post Weld Treatments

The first stage of post weld treatment employed for the study was conventional heat treatment. Nitrogen (Brother gas, Dubai, UAE) was used as cooling media for tempering process similar to the work done by Prieto et al. [12]. The heat treatment cycle is as follows: heating at a rate of 150 °C/h up to 740 °C with a holding time of 15 min and cooling to room temperature at a rate of 200 °C/h. This specimen is defined as HT specimen. To improve the mechanical properties of dissimilar welds, a subzero temperature treatment is carried out after heat treatment cycle. In addition to heat treatment cycle, Deep Cryogenic Treatment (DCT) at −195 °C with a soaking time of 15 min was performed and brought to room temperature. This specimen is identified as CT specimen. The cooling rate was chosen in such a way that the entire treatment was carried out for 9 h. The welded specimens without any treatments is defined as UT specimens.

## 4. XRD Analysis

**Figure 3:**
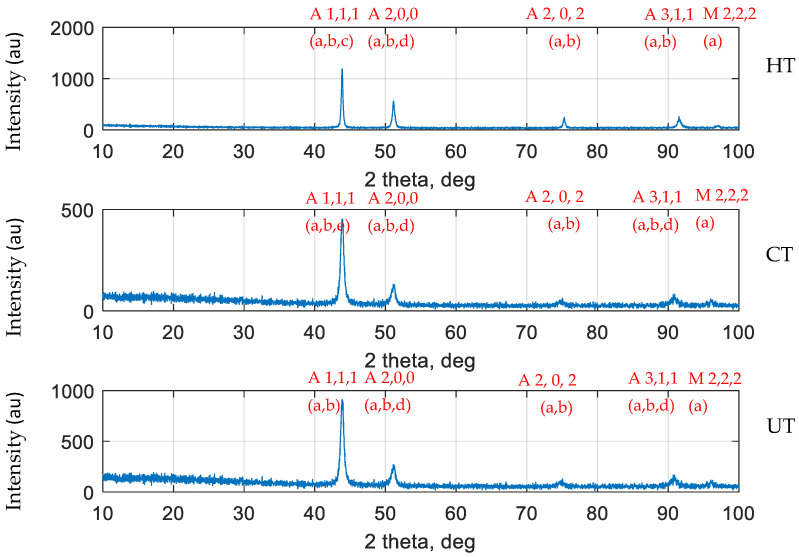
XRD failure region analysis for all specimens at SS 316L HAZ.

HAZ zone of SS 316L base metal was observed as the failure region for all samples (Table S1—Supplementary Material). XRD patterns (PANalytical X’pert-Pro Diffractometer, Malvern, UK) of Heat Treated (HT), Cryo-treated (CT), and Untreated (UT) specimens are shown in Figure 3. The austenite peaks were identified at 43.69°, 44.31°, 43.51° angle (2θ) for HT, CT, and UT specimens, respectively. It was also observed that the peak intensity of CT was lower when compared to HT and UT specimens. The phase correlation carried out on the 2θ peaks for HT, CT, and UT specimens has resulted in Cr_23_C_6_, FeNi in general. This phenomenon was noticed in all specimens and however it is predominant in CT specimen. In addition, CT specimen has correlated to Fe_3_ C phase also. The miller index identified for all specimens were the same. Minor shift in major peak has been observed for CT specimen when compared to HT and UT specimens.

## 5. Micro Hardness Distribution

**Figure 4:**
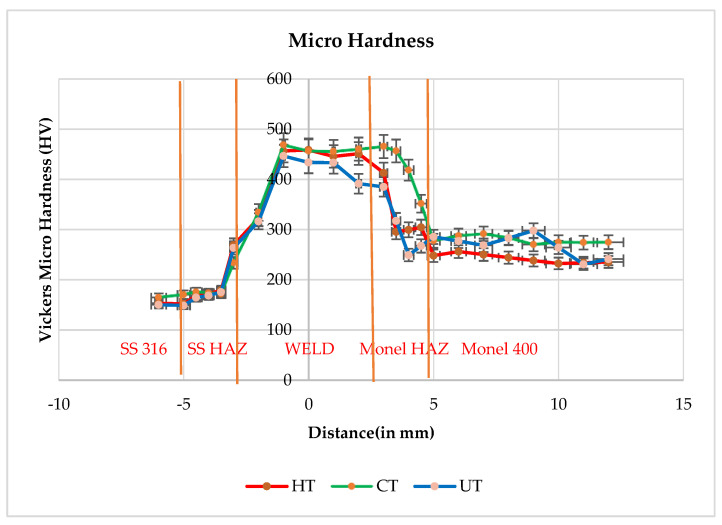
Micro hardness mapping of zones for HT, CT, and UT.

As shown in the Figure 4, for micro hardness mapping (Matsuzawa Co.,Ltd, Toshima, Japan) of HT, CT, and UT specimens, higher hardness values were observed in SS 316L HAZ when compared to SS 316L base and it is close to the respective hardness values of Monel 400 HAZ and Monel 400 parent metal. It was noticed that the weld zone hardness was much higher that of parent metal SS 316L (440 to 460 HV) for all the cases. Yuan-Zhi Zhu et al. [13] reported that the increase in hardness is attributed to the transformation from austenite to martensite and the precipitation of tiny carbides. The presence of carbides are addressed in XRD results. Micro hardness measurements showed the highest values in the weld, the lowest in HAZ from the SS 316L stainless steel side. This distribution is a consequence of structural changes caused by the influence of the welding thermal cycle [14].

## 6. Tensile Test, Digital Image Correlation, Fractography, and Dimple Size Analysis

**Figure 5:**
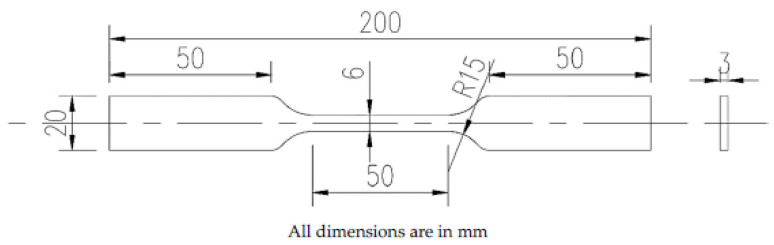
Tensile specimen dimensions.

The tensile test specimens were prepared as per ASTM E8 [15] (Figure 5) and tests were conducted with three replications and the average values of strength and % elongation was used for further study. Shimadzu servopulser 100 kN dynamic testing machine (Shimadzu Corporation, Kyoto, Japan) with SFL-100kN-B load cell was used for tensile testing at the rate of 0.1 mm/s. 

### 6.1. Comparison of Tensile Test Results of UT, HT, and CT Specimens 

**Figure 6:**
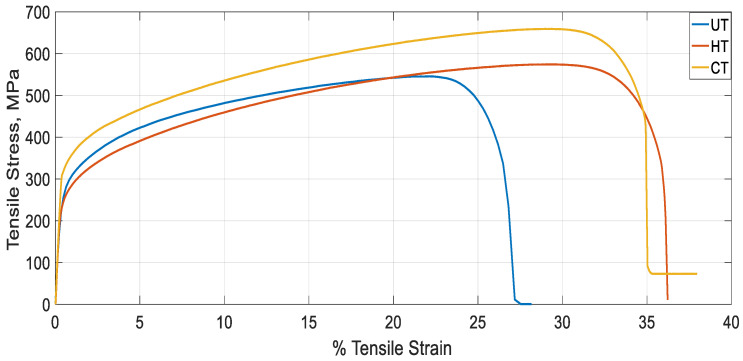
Engineering stress strain graph for HT, CT, and UT specimens.

Area under the stress strain curve (Figure 6) depicts the energy absorbed by the material prior to failure and area under this curve up to elastic limit is the modulus of resilience. To have a larger area, materials should have a good compromise between ductility and strength. CT specimen has higher toughness in comparison with UT and HT specimens with UT specimen having lowest toughness. Hence, it can be inferred that, CT specimen has higher ability to store or absorb energy without permanent deformation in comparison with UT and HT specimens. Zakaria Boumerzoug et al. [16] suggested that, the changes in mechanical properties across the weld may be due to several factors such as residual stresses, grain size, phase composition, and metallic inclusions. Since this phenomenon is more pronounced during post weld treatments, variations in experimental results were noticed. Table 4 represents the tensile properties for UT, HT, and CT specimens tested. The highest value of mechanical properties in terms of yield strength (263.43 MPa) and tensile strength (659.11 MPa) were observed in CT specimen. On the other hand, the lowest value of yield strength (186.06 MPa) and lowest value of tensile strength (545.49 MPa) were obtained in the UT specimen. The presence of carbide precipitates in CT and HT specimens would have resulted in better performance of those specimens when compared to UT specimen. Touseef Nauman et al. [17] stated that CT aided residual stress relief, conversion of retained austenite to martensite, and refinement of carbides. From Figure 6, fracture stress is close to zero for UT and HT specimens and it is close to 100 MPa for CT specimen which points to good elastic behavior in all the cases. The failure occurred for all the specimens near SS 316L HAZ region and the fracture surface resembled ductile mode of fracture.

### 6.2. Digital Image Correlation (DIC)

DIC was used to analyze the strain distribution on dissimilar metal weld which is a heterogenous combination of different materials. The current study involves determination of the displacement and strain values of GTAW of dissimilar weld joint on Monel 400 and SS 316L under tensile loading using Ncorr 2D digital image correlation MATLAB program (Version 1, Georgia Institute of Technology, Atlanta, GA, USA) for HT, CT, and UT specimens [18].

#### 6.2.1. DIC Specimen Preparation

**Figure 7:**
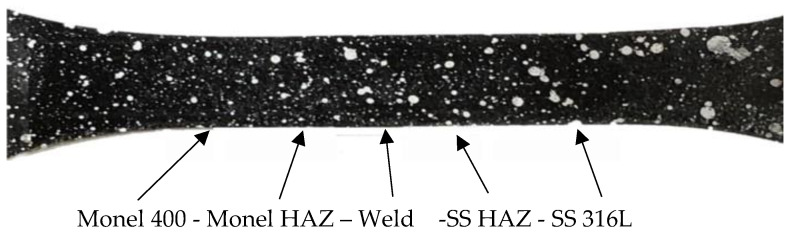
Speckle pattern on specimen.

Tensile test specimen surface was subjected to surface grinding machine (CCCP, Moscow, Russia) since smooth surface will increase the accuracy of DIC method. Specimens were painted black (Jotun, Dubai, UAE) initially and white spray paint (Jotun, Dubai, UAE) is used to spray a speckle pattern on the specimen at a distance. Speckle pattern is the random dots of white paint on black surface of specimen as shown in Figure 7. Tensile testing for GTAW specimens of HT, CT, and UT are captured using Charge Coupled Device (CCD) camera (Nikon Corporation, Tokyo, Japan) at a rate of 2 fps. The specimen is properly inserted on the machine to avoid misalignment. CCD camera is focused according to area of the focus which is the gauge length of the specimen. Image resolution defines accuracy of the results. Once test started, camera controlling software took photos at regular interval with automatic focus adjustment. Images at the interval of 5 s were considered for the analysis and 5 stages at regular intervals were used for comparison of results.

#### 6.2.2. Analysis Using Ncorr 2D Digital Image Correlation

**Figure 8:**
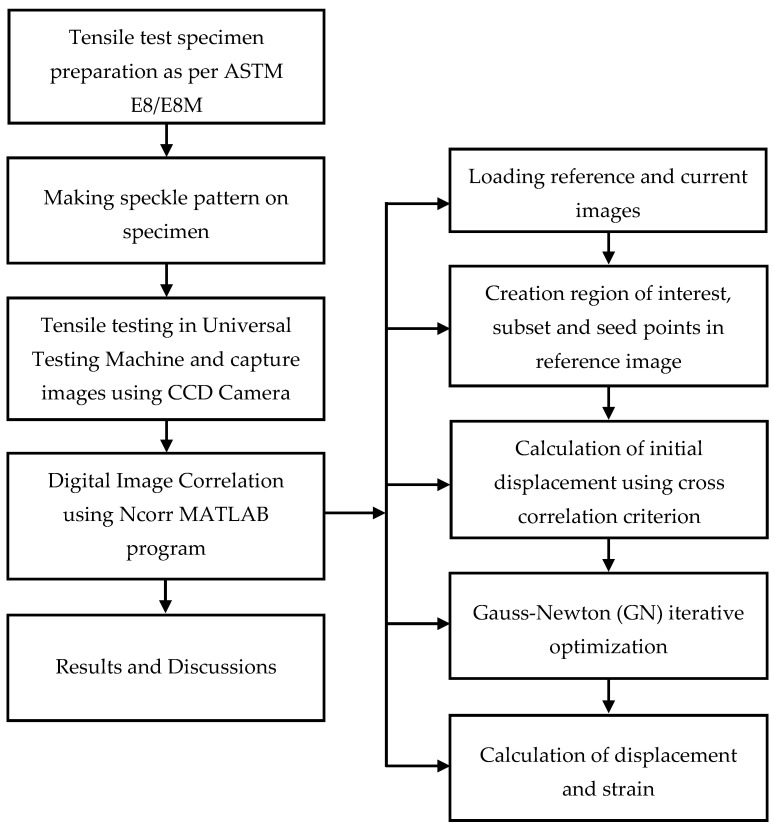
Flow chart for DIC analysis.

Tensile test images were analyzed using Ncorr MATLAB program to find displacement and strain distribution. The main DIC algorithm used in Ncorr is based on Bing Pan’s Reliability Guided—Digital Image Correlation framework which is more robust [19]. DIC does this by taking small subsections of the reference image, called subsets and determining their respective locations in the current configuration. Reference image (first image before deformation) was uploaded initially and subsequently other images at various instances of loading were uploaded. Middle portion of the specimen region was considered for DIC analysis. The subsets are tracked in reference and deformed (current) images through temporal matching and correlation functions. A correlation coefficient is used to analyze how each subset has moved and deformed during test and match the similarity between the subsets in deformed (current) and non-deformed (reference) images. After setting subset value, seed points in the analysis region are defined. Seed points provide initial solution for the Reliability Guided – Digital Image Correlation analysis. An initial solution is required for an iterative optimization scheme converge to a local maximum and minimum. Based on correlation coefficient the initial solution is calculated using fast normalized cross correlation method. Initial solution of displacement values from cross correlation criterion are optimized by Gauss-Newton (GN) iterative optimization scheme. The displacement for each subset is calculated and each subset is repeated over the complete surface. This results in displacement map of over the complete surface of the specimen. Using a calibration image by setting a line across the specimen unit is converted from pixel to mm. Green-Lagrangian and Eulerian-Almansi strains are calculated from the displacement data by using a least squares plane fit to a local group of data points. DIC Analysis of TIG Welded Tensile Test Specimen using Ncorr MATLAB Program workflow is shown in Figure 8.

#### 6.2.3. DIC Results and Discussion

Filler wire material (ENiCrFe-5) has higher tensile strength compared to Monel 400 and SS 316L and Monel 400 has 6.8% more strength than SS 316L. Hence SS 316L is the weakest zone in the welded specimen. For all the specimens, which were subjected to tensile loading, failure occurred at SS 316L alloy region. In DIC, the location of dissimilar weld was divided into 5 different zones Monel 400 base, Monel 400 HAZ, Weld metal, SS 316L HAZ, SS 316L base for HT, CT, and UT specimens. The true stress-strain relationship [20] is given by using Equation (1).

(1) $$\sigma_{i}\;=\;\frac{P_{i}}{A_{i}}$$

*P_i_* and *A_i_* are instantaneous tensile load and cross-sectional area, respectively, and instantaneous cross-sectional area *A_i_* is given by Equation (2).

(2) $$A_{i}\;=\;A_{0}e^{\left(-\varepsilon_{i}\right)}$$

**Figure 9:**
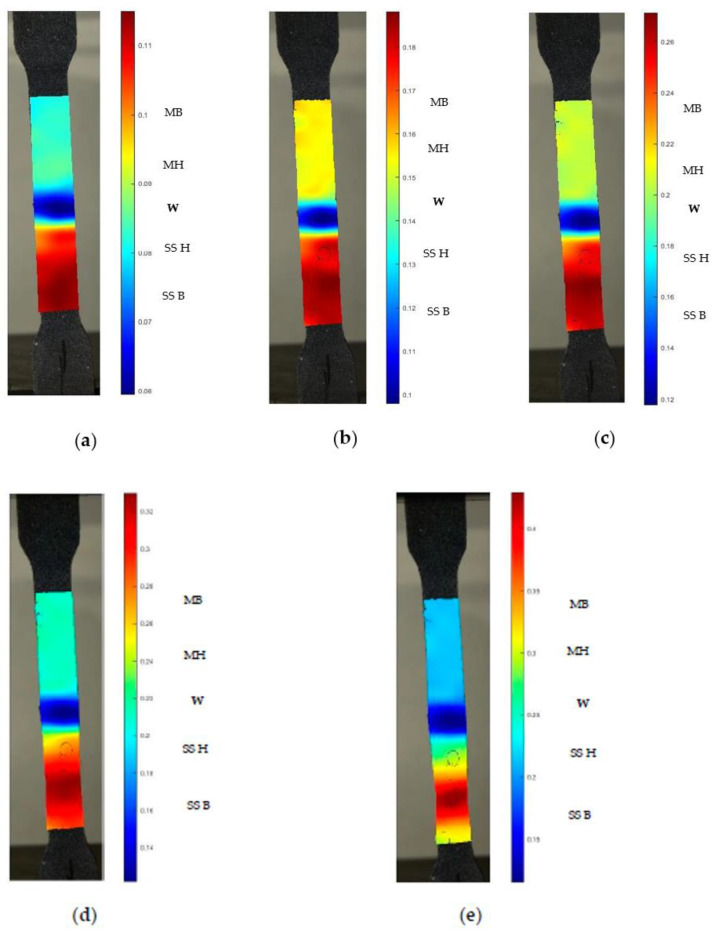
Strain distribution across weld specimen during a tensile test for CT specimen: (**a**) stage 1; (**b**) stage 2; (**c**) stage 3; (**d**) stage 4; (**e**) stage 5; MB–Monel 400 Base, MH–Monel 400 HAZ, W–Weld, SS H–SS 316L HAZ, SS B–SS 316L Base.

In Equation (2), *A*_0_ is the initial cross-sectional area and *ε* is the major strain in a local zone estimated by DIC analysis [20]. At each stage of CT specimen, local strain distribution was predicted from DIC and shown in Figure 9. Around 250 images were taken for each specimen and five stages were considered for the analysis. The local strain values are predicted for five stages from loading to fracture. Strain distribution for CT specimen for each stage is consolidated in Table 5.

The elongation of CT specimen in different stages is shown in Figure 9. At initial stage, maximum local strain (11.5%) was observed at SS 316L HAZ compared to Monel 400 and weld region due to lesser tensile strength of SS 316L. Strain at SS 316L HAZ region is 6.08% more than SS 316L base due to formation of residual stress. Weld zone has lesser strain compared to Monel due to its higher tensile strength. Compared to Monel 400 base, Monel 400 HAZ has lesser strain. Similar trend was observed in all the five stages. Stage 5 represents necking region of CT specimen. Maximum strain was observed for SS 316L HAZ due to grain coarsening. In-homogeneous strain distribution has been observed and strain localization was observed in SS 316L HAZ which resulted in necking. The strain at 5th stage at SS 316L HAZ of the weld joint is over 44.31%, while the strain at weld zone was only 11.4%. This suggests that the tensile failure may occur in the welded joint as a result of local damage behavior due to in-homogeneous strain distribution [11]. Similar observations were made for HT and UT specimens.

**Figure 10:**
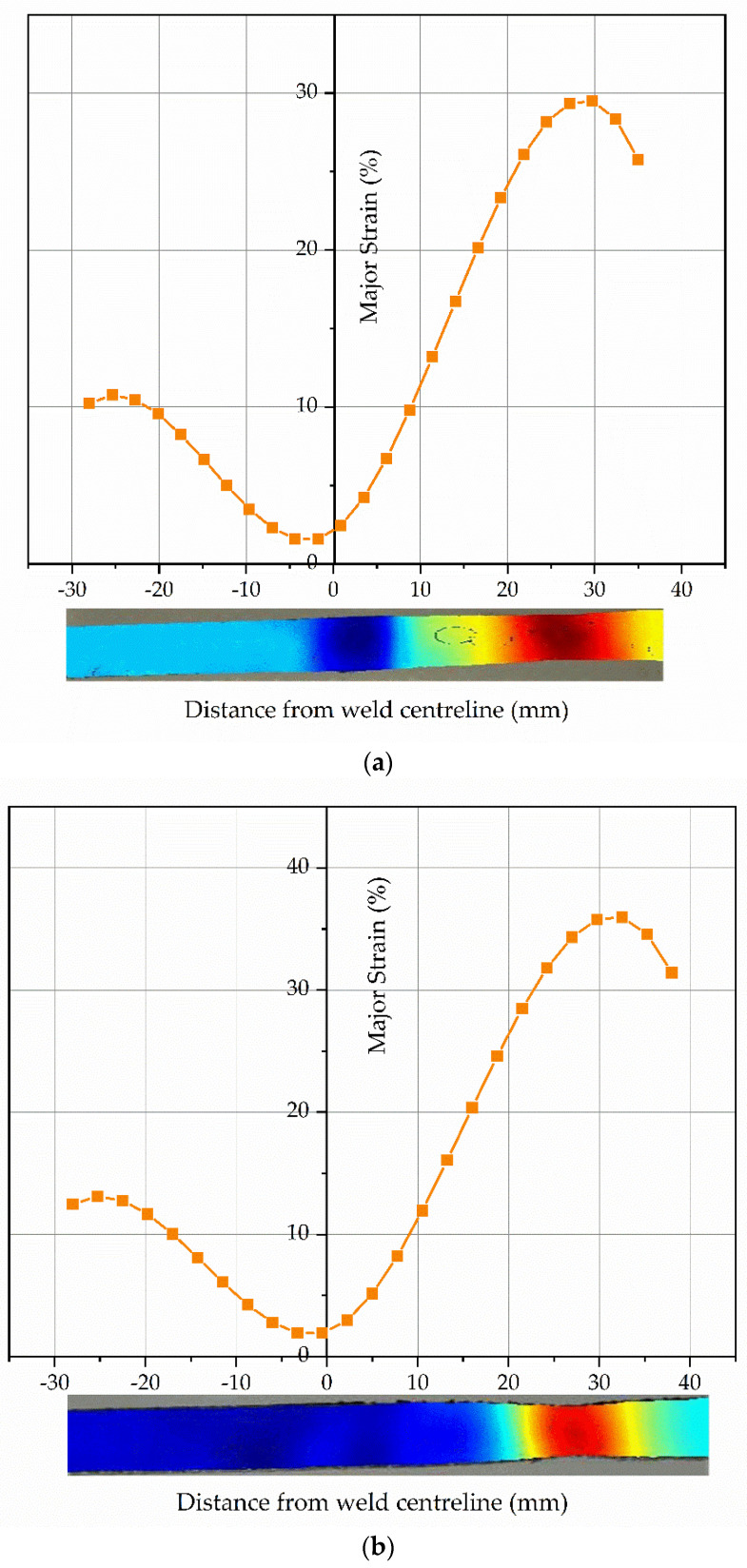

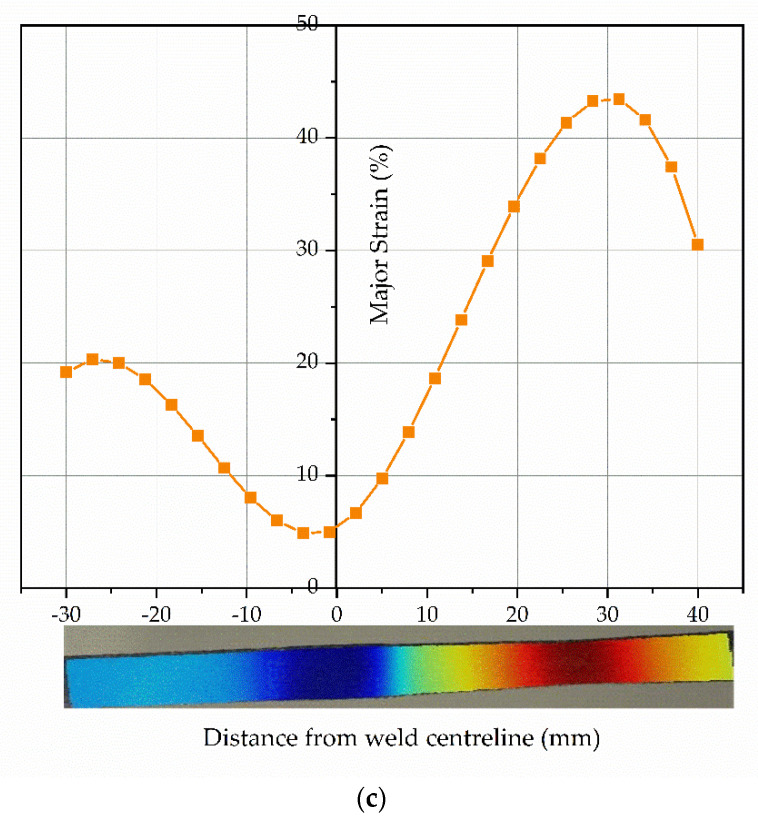
Strain distribution prior to fracture during DIC analysis: (**a**) UT specimen; (**b**) HT specimen; (**c**) CT specimen.

The color patterns of strain distribution were found to be different for different specimens. Localized strain on HAZ for SS 316L rises with the increase in stress for all cases. The strain distribution at necking region prior to fracture for UT, HT, CT specimens along distance from weld center line was predicted and presented in Figure 10. The local strain for all specimens before yielding on both sides of the weld centerline remains almost symmetrical due to equivalent young’s modulus. The local strain for all specimens after yielding on both sides of the weld centerline remained asymmetrical. This may be due to difference in tensile strength of the different materials [20]. Monel base metal and weld fusion zone (WZ) have higher yield strength values as independent materials. The dissimilar weld in tensile loading shows lower strain distribution on these zones as shown in Figure 10. Subsequently, the necking occurred at region of SS 316L Base Metal (BM) closer to SS 316L HAZ side in the 5th stage.

**Figure 11:**
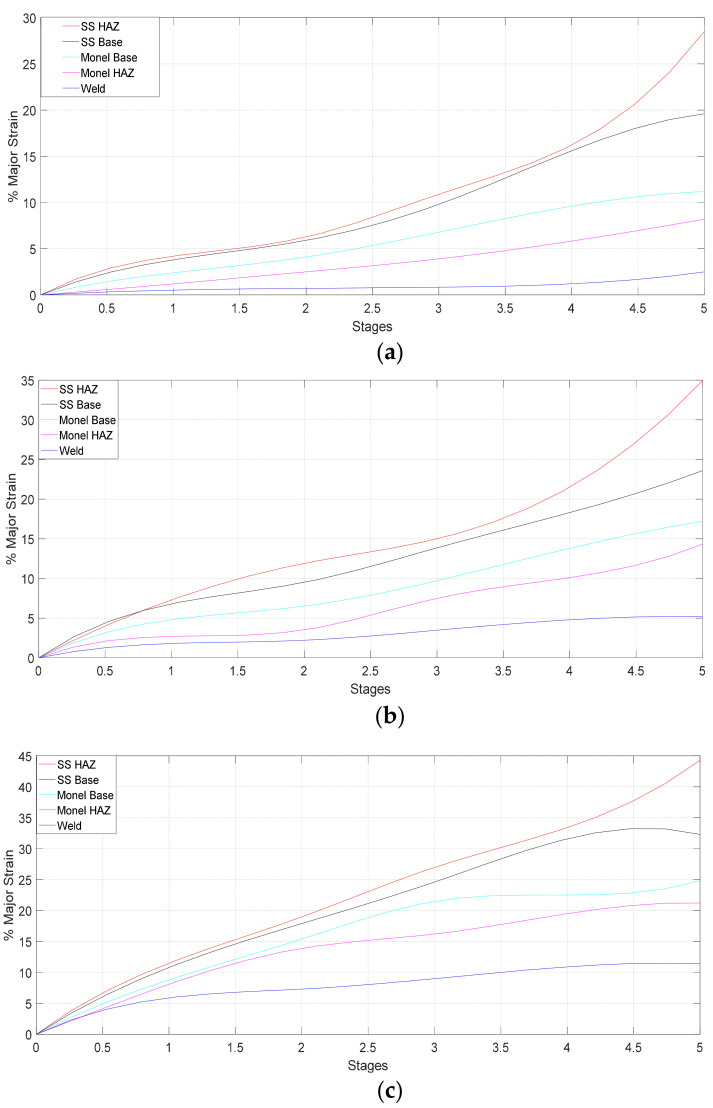
Variation of local strain at different regions of the weld: (**a**) UT specimen; (**b**) HT specimen; (**c**) CT specimen.

Variation of major strain for UT, HT, and CT specimens at different stages are obtained from the DIC results and plotted in Figure 11. In all cases, strain has increased linearly up to certain stage (yield point) after that it decreased slightly and again increased up to necking. This suggests that the elastic deformation, as well as plastic deformation, have occurred in the specimens. Strain is more pronounced in SS 316L HAZ for all specimens. Maximum local strain values for UT, HT, and CT during necking were found to be 29.5, 35.96, and 44.31%, respectively. CT specimen has 18.84 and 33.42% higher strain values when compared to HT and UT specimens. It can be concluded that, the ductility of the weld joint has been improved by the cryogenic treatment which has resulted in larger strain values in all the zones.

**Figure 12:**
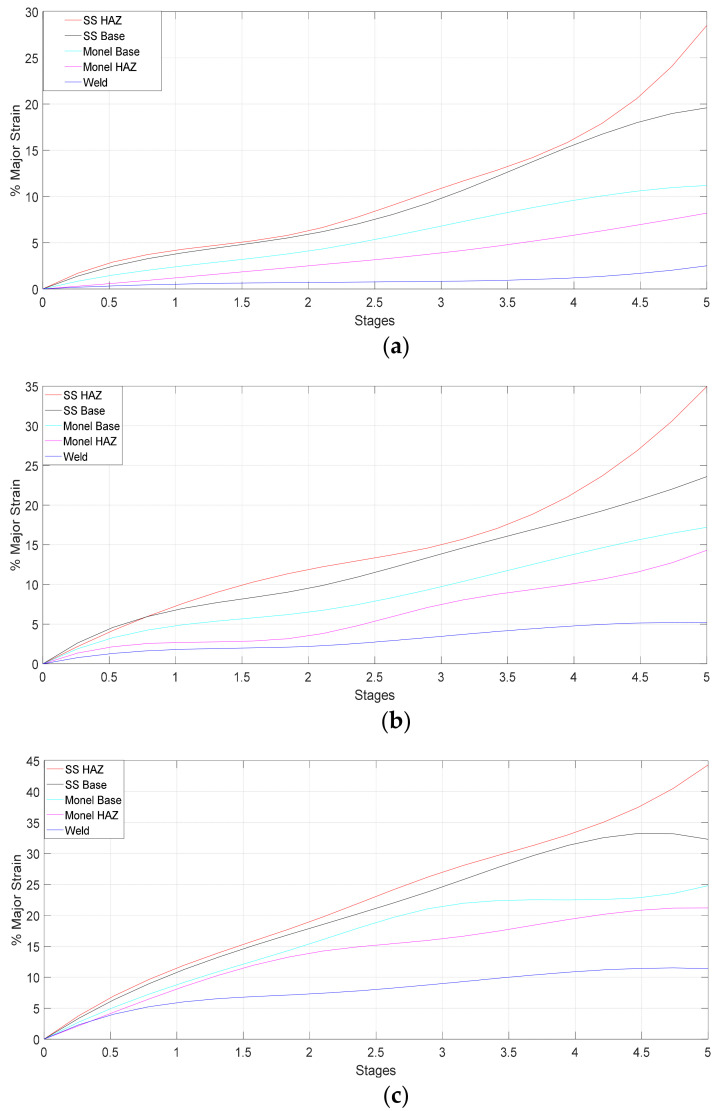
Local stress strain curves of different zones of the weld joint: (**a**) UT specimen; (**b**) HT specimen; (**c**) CT specimen.

Local stress-strain behavior of different zones for UT, HT, and CT specimens are predicted using Equations (1) and (2) are shown in Figure 12. The local stress-strain curves displayed higher yield strength values for locations on weld, Monel 400 base and Monel 400 HAZ. On the other hand, SS 316L HAZ and its base metal showed lower yield strength values locally. For most part of the plastic zone, the highest stress and strain values were observed in SS 316L HAZ. The local stress-strain curves of individual weld zones provide a clear indication of the heterogeneity of the local mechanical properties [11]. For all cases, CT has higher true stress (1155.16 MPa) compared to HT (988.91 MPa) and UT (875.42 MPa) specimens. CT specimen has better strength and ductility compared to other specimens.

### 6.3. SEM for Tensile Specimens 

The HT, CT, and UT welded specimens considered for the study have satisfactory tensile properties and the weld strength was found to be much better than both the parent metals. CT specimen exhibited better tensile and yield strength values when compared to other specimens. Additionally, the tensile test results match well with the hardness data. SEM fractographs (Tescan VEGA3 XMU, Kohoutovice, Czech Republic) of the tensile tested samples of UT, HT, and CT dissimilar weldments with ERNiCrfe-5 filler were carried out. Fractography studied on UT, HT, and CT specimens experienced failure at the parent metal HAZ of SS 316L or between HAZ and fusion zone in all cases. All specimens showed basic features on the fracture surface which are commonly noticed in ductile fracture. This fracture surface consists of dimples which show failure in a ductile manner and demonstrated characteristic of cup-cone shaped fracture type which was also reported by Buddu R. K et al. [21], for stainless steel. Nucleation, growth and coalescence of voids result in dimples resulting in crack growth. Beachem C.D. et al. [22] have stated that, dimple size varies widely from micrometer to nanometer based on the process and material parameters.

#### 6.3.1. SEM Observation of UT Specimen

**Figure 13:**
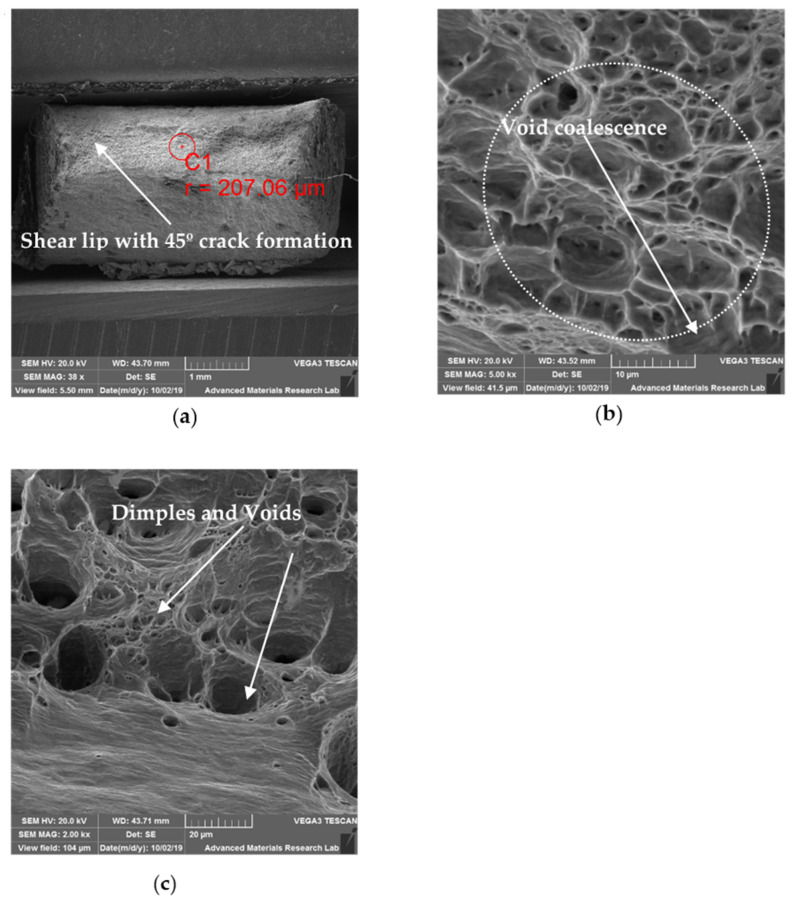
SEM fractography images of UT specimen: (**a**) 35×; (**b**) 2000×; (**c**) 5000×.

Under identical condition of strain loading, HAZ of austenitic stainless steels (SS 316L) commonly experience ductile failure governed by dislocation flow, their mutual interactions, and interactions with other second-phase particles and phases as reported by Ram Kumar et al. [23]. Figure 13a–c shows the SEM images of UT specimen at different magnifications. The images show the presence of ductile failure which are in line with the tensile test results. The phenomenon of shear fracture after necking was observed in Figure 13a. The shear lips are more distinct when compared to HT and CT specimens. Coalescence of micro-voids is noticed at higher magnification and the mixed mode of fracture is evident with increased area of facet when compared to HT and CT specimens (Figure 13b,c).

#### 6.3.2. SEM Observation of HT Specimen

**Figure 14:**
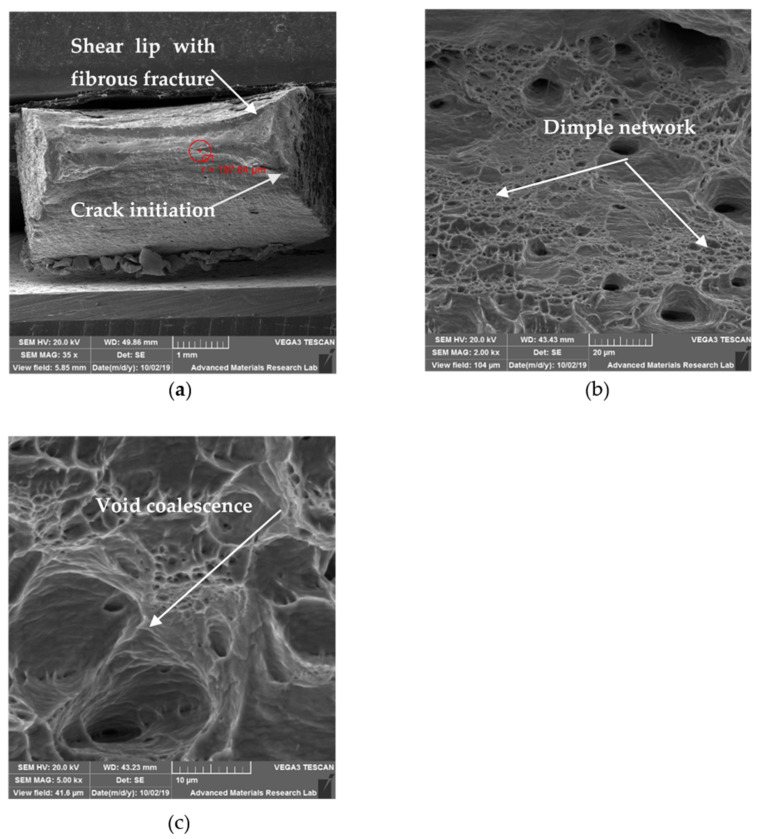
SEM fractography images of HT specimen: (**a**) 35×; (**b**) 2000×; (**c**) 5000×.

Post weld treatments normally result in homogenization of microstructure in welds and improve mechanical properties. Post weld heat treatments in austenitic stainless steel will result in formation of Cr and other alloying elements related carbides which may result in depletion of such alloying elements. The SEM fractography observed for HT specimen is shown in Figure 14a–c. At low magnification, it can be noticed that, the ductile fracture would have started from center of neck region and moved outward. Fracture propagated approximately at 45° in the transverse direction. This type of fracture normally results in the formation of shear lip which was also evident in Figure 14a. The fibrous matte surface noticed on the fracture surface suggests ductile nature. At 5000×, the dimple network is noticed along with void coalescence (Figure 14b). According to Pengfe Li et al. [24], the dimple structure denotes ductile fracture and if it is in excess, it may lead to reduction in tensile strength. At 2000× magnification along with dimple structure, flat regions were observed suggesting mixed mode of fracture (Figure 14c).

#### 6.3.3. SEM Observation of CT Specimen

**Figure 15:**
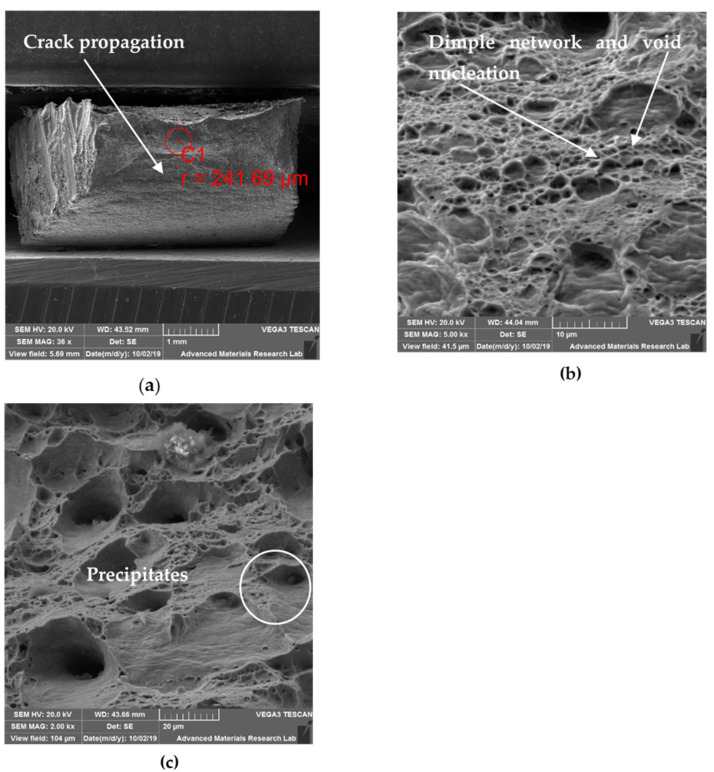
SEM fractography images of CT specimen: (**a**) 35×; (**b**) 2000×; (**c**) 5000×.

Singh T.P. et al. [25] stated that tempered post cryogenic treatment of boron steel on post tempering resulted in grain coarsening and martensite decomposition with bubble and dimples coalescence along the grain boundaries. The fracture surfaces exhibited a mixed morphology with micro voids and elongated dimples which are characteristic representation of ductile fracture. Figure 15a shows the crack propagation of the fractured specimen at lower magnification. The shear lips are not well defined, and the crack propagates along the shear lips. Figure 15b shows micro dimples due to plastic deformation which is the evidence for higher energy absorption before failure. Images also displayed elongated voids bordered with the fibrous network which are phenomena for representing strengthening characteristics, as reported by Malhotra et al. [26]. Robert et al. [27] who studied the CT austenitic stainless steel fracture behavior has stated that the fracture path is mixed, through inter and intra particles and in the same area very different behaviors can coexist. Figure 15b shows the presence of mixed mode of fracture and Figure 15c shows the presence of second phase particles in the fracture zone. Alireza Khalifeh et al. [28] and S. P. Lynch et al. [29] have stated that, precipitation hardening cause strain localization initiating void nucleation along the grain boundaries which would result in inter granular fracture as noticed in Figure 15b.

### 6.4. Dimple Size Analysis Using Image Processing

**Figure 16:**
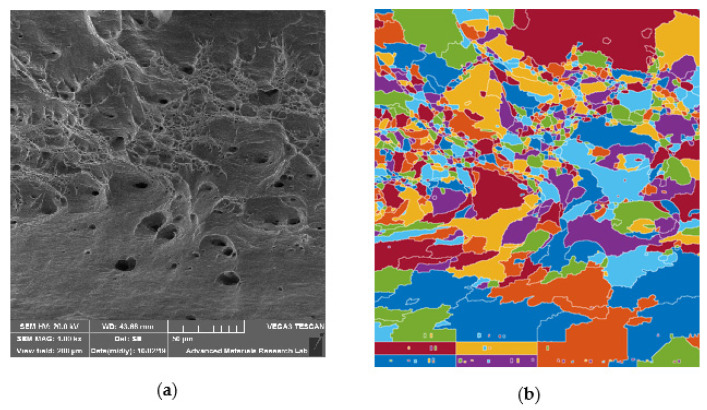
Dimple size analysis for UT specimen: (**a**) SEM image at 1000×; (**b**) Processed image.

**Figure 17:**
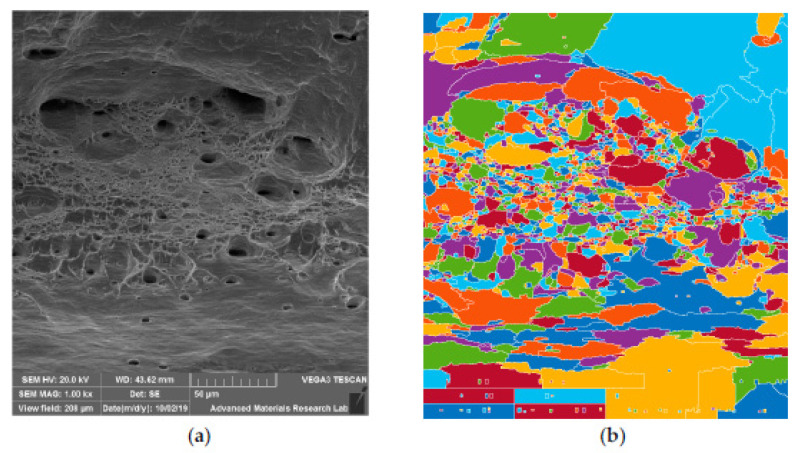
Dimple size analysis for HT specimen: (**a**) SEM image at 1000×; (**b**) Processed image.

**Figure 18:**
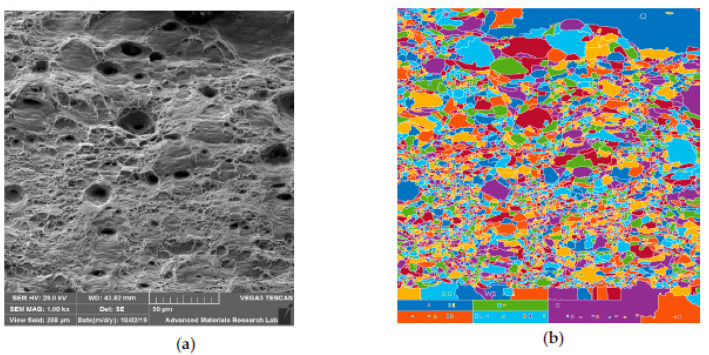
Dimple size analysis for CT specimen: (**a**) SEM image at 1000×; (**b**) Processed image.

The physical process of ductile fracture of specimens UT, HT, and CT is mostly recognized to be based on a sequence relating to void nucleation, void growth, and void coalescence importunately to micro crack formation. The process of ductile fracture comprising void nucleation and growth are principally influenced by the type of dislocation and dislocation interactions which are mainly administrated by the state of strain hardening of the dissimilar weld during the weld heating and cooling of HAZ [30]. For better understanding of this phenomena, the entire range of dimples has to be analyzed for size and density distribution. Image processing using Matlab sotware (R2016b, MathWorks, Natick, MA, USA) is a dominant tool for distinguishing the void morphologies on fracture surfaces. Dimples within a fracture surface can easily be detected by image processing due to the high degree of contrast between the colors and the more reflective dimples at its peripheries. The size of small dimples in the fracture is related to the size of grains. While large dimples, which had a more complex shape, resulted from the coalescence of voids, the results obtained were typical of high-strength materials characterized by a significant number of small dimples and only a few large dimples. Such distribution of dimples demonstrates a combination of high strength and sufficient plasticity. The dimple densities and sizes were analyzed in Figure 16, Figure 17 and Figure 18 indicative of ductile rupture. With the change in post weld heat treatment, dimple size and dimple density, varied significantly and a comparison between different specimens is made as shown in Table 6.

Higher dimple density and reduction areas were observed in cryotreated sample CT in comparison with heat treated (HT) specimens of dissimilar weld. Arpan Das et al. [31] states that, that lower mean dimple sizes indicate higher strength of the specimens which are observed in CT specimen, followed by HT and UT specimens. The circularity values are in the range of 0.641 to 0.739 suggesting elongation in all cases. Failure in the adjacent region of the weld (HAZ) of SS 316L has happened in a region with less hardness in which grain growth and carbon reduction were observed and the cryotreated (CT) samples exhibited higher ductility than UT and HT samples.

**Figure 19:**
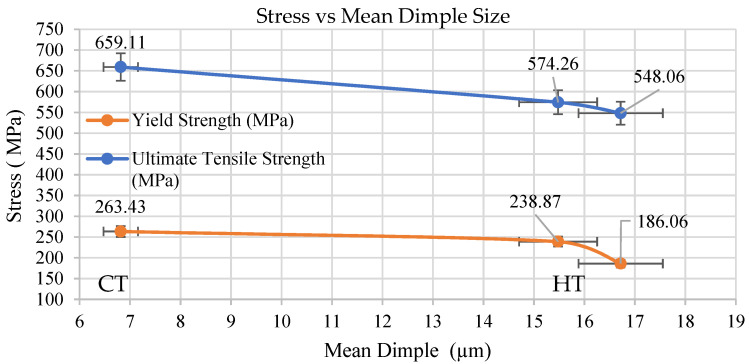
Mean dimple size vs stress.

**Figure 20:**
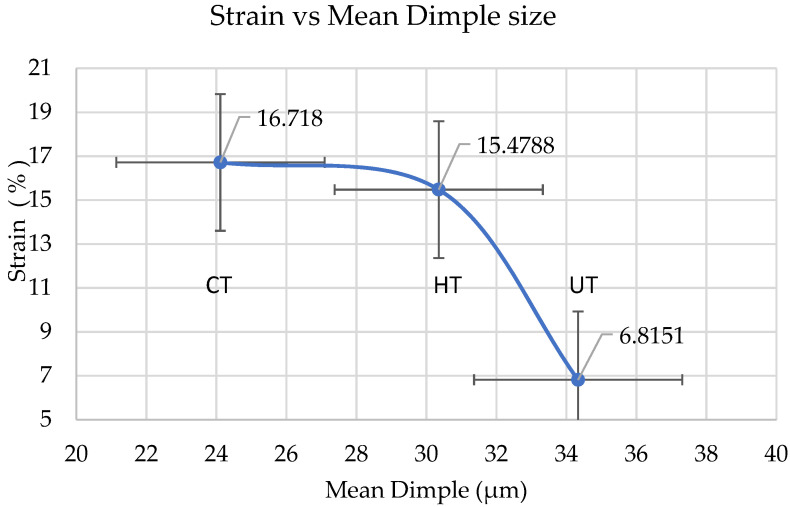
Mean dimple size vs % elongation.

The effect of mean dimple size on stress and elongation was predicted. As seen from Figure 19, the tensile strength is found to be more for CT specimen when compared to HT and UT specimens which was found to be inversely proportional to the mean dimple size. Similarly % of elongation for CT specimen was found to be more (Figure 20) which may be attributed to the formation of deformation induced martensite (DIM) in certain austenitic stainless steels and it this will result in void nucleation to promote further deformation. Mehmet Erdogan et al. [32] have reported that controlled martensite formation would increase both ductility as well as strength. The presence of martensite improves strengthening and at the same time, it creates nucleation sites to increase ductility. Similar observations where martensite promotes void nucleation are also reported by P. Poruks et al. [33], in a low-carbon bainitic steel.

## 7. Conclusions

Monel 400 and SS 316L dissimilar metal welding was carried out using GTAW and joints were subjected to post weld treatment and deep cryogenic treatment. Tensile testing was carried out experimentally and digital image correlation was performed to analyze strains at different zones of weld region. Scanning Electron Microscopy was used to analyze the fracture surface. 

1. CT specimen has more ultimate strength and % Elongation compared to HT and UT specimens. CT specimen has 16.48 and 12.87% more tensile strength when compared to UT and HT specimens. The % Elongation of CT was 27.81 and 13.73% more than UT and HT specimens, respectively.

2. The local strain values are predicted for five stages from loading to fracture for UT, HT, CT specimens using DIC. In CT, Maximum strain value of 44.31% was observed for SS 316L HAZ. The deformation was localized rapidly at the necking region on the SS 316L HAZ side until failure. Similar effects were noticed in HT and UT specimens also.

3. The strain distribution at necking region for UT, HT, CT specimens along distance from weld center line was predicted. The local strain for all specimens before yielding on both sides of the weld centerline remains almost symmetrical due to equivalent Young’s modulus. The local strain for all specimens after yielding on both sides of the weld centerline remains asymmetrical due to different tensile strength.

4. Variation of major strains for UT, HT, and CT specimens at different stages are obtained and plotted graphically. In all cases, strain increases linearly up to certain stage (yield point) after that it decreases slightly and again increases up to necking. Elastic as well as plastic deformation has occurred in all the specimens. Maximum local strain values for UT, HT, and CT prior to fracture were found to be 29.5, 35.96, and 44.31%, respectively, in SS 316L HAZ.

5. Local stress-strain curves of different zones for UT, HT, and CT specimens are predicted and plotted graphically. Higher yield strength values for locations on Weld, Monel 400 base and Monel 400 HAZ were noted while SS 316L HAZ and its base metal showed lower yield strength values locally. For most part of the plastic zone, the maximum stress and strain were observed in SS 316L HAZ and necking occurred at region of Stainless Steel -BM closer to SS HAZ side until failure. For all cases, CT has higher true stress (1155.16 MPa) compared to HT (988.91 MPa) and UT (875.42 MPa) specimens. CT Specimen has better strength and ductility compared to other specimens.

6. From the dimple size analysis on the fracture surface using image processing, it is observed that CT specimen has smaller dimple size and higher density when compared to UT and HT specimens. Hence the ductility as well as strength of the CT specimen is better than UT and HT specimens.

The present study has limitation in terms of process parameters considered. Further analysis is required for optimization of weld bead geometry and cryogenic treatment related parameters and selection of alternative filler wires.

## Figures and Tables

**Table 1:** Chemical composition.

% Wt	C	Cr	Fe	Mn		P	S	Si	Mo	Cu	Co	Ni
SS 316L	0.03	17	65.64		2	0.045	0.03	0.75	2.5	0	-	12
Monel 400	0.3	-	2.5		2	-	0.024	0.5	-	31.6	-	63
ENiCr Fe-5	0.04	16	6–10		1.0	0.03	0.015	0.35	-	0.5	0.1	70

**Table 2:** Material properties.

Properties	Monel 400	SS 316L	AWS*- ERNiCrFe-5
Modulus of Elasticity	179 GPa	193 GPa	190 GPa
Tensile Strength (Annealed)	550 MPa	515 MPa	630 MPa
Yield Strength (Annealed)	240 MPa	205 MPa	-
Elongation	48%	60% (in 50 mm)	34%

*AWS—American Welding Society

**Table 3:** Welding parameters.

Parameter	Value of the Parameter
Filler wire	ENiCrFe-5
Current, Amps	120
Gas flow rate, LPM	16
Welding speed, mm/s	3.5
Heat input, KJ/mm	0.4114
Groove angle	V-type −60°
Bevel angle	30°
Plate thickness, mm	3
Root face thickness, mm	1
Root opening, mm	2
Polarity	DCEN
Backing gas (Argon), lpm	5 to 7
Tungsten size and type	1/8”, 2% throated tungsten

**Table 4:** Experimental results of tensile testing.

Specimens	Yield Strength (MPa)	Ultimate Strain (%)	Ultimate Tensile Strength (MPa)
UT	186.06	24.81	548.06
HT	238.87	29.65	574.26
CT	263.43	34.37	659.11

**Table 5:** Strain distribution for CT specimen in each stage.

Local Strain (%)					
Stage	Monel 400 Base	Monel HAZ	Weld	SS 316L HAZ	SS 316L Base
1	8.8	8.1	5.9	11.5	10.8
2	15.4	13.9	7.3	18.96	17.9
3	21.5	16.2	8.98	26.99	24.6
4	22.5	19.5	10.9	34.4	31.6
5	24.8	21.2	11.4	44.31	32.3

**Table 6:** Dimple sizes for UT, CT, and HT specimen.

Specimen	Min, µm	Max, µm	Mean, µm	Count	Density, µm^2^	Circularity
UT	1.6546	185.980	16.718	200	0.010087	0.641
CT	0.1653	41.042	6.8151	865	0.035161	0.683
HT	0.3283	70.918	15.4788	572	0.019383	0.739

## Data Availability

No new data were created or analyzed in this study. Data sharing is not applicable to this article.

## Supplementary Materials

The following are available online at https://www.mdpi.com/1996-1944/14/6/1560/s1, Table S1: XRD data for failure region analysis of all specimens at SS 316L HAZ.

## Nomenclature and Abbreviation

*σ_i_*	true stress
*ε_i_*	true strain
*A*_0_	initial cross-sectional area
*A_i_*	instantaneous cross-sectional area
*P_i_*	instantaneous tensile load
AISI	American Iron and Steel Institute
ASTM	American Society for Testing and Materials
CCD	charge coupled device
CT	Heat treated followed by cryogenic treated specimen
DCT	deep cryogenic treatment
GTAW	gas tungsten arc welding
DIC	Digital Image Correlation
HAZ	heat affected zone
BM	Base metal
HT	heat treated specimen
UT	Untreated specimen
