# Supplementary material for: Digital Image Correlation of Tensile Properties for Monel 400/SS 316L Dissimilar Metal Welding Joints
Source: Materials (Basel). 2021 Mar 22;14(6):1560. doi: 10.3390/ma14061560 (PMC8005209; doi:10.3390/ma14061560)
Supplement: Supplementary file 1 [file materials-14-01560-s001.pdf]

materials MDPI

Supplementary

Digital Image Correlation of Tensile Properties for MonelAlloy 400/SS 316L Dissimilar Metal Welding Joints

Cherish Mani [1,*], Sozharajan Balasubramani [1], Ram Karthikeyan [1,*] and Sathish Kannan [2]

[1] Department of Mechanical Engineering, Birla Institute of Technology & Science, Pilani, Dubai Campus, University, Dubai P.O. Box 345055, United Arab Emirates; Sozharajanram@gmail.com
[2] Department of Mechanical Engineering, American University of Sharjah, Sharjah P.O. Box 26666, United Arab Emirates; skannan@aus.edu
* Correspondence: cherishmani@gmail.com (C.M.); rkarthikeyan@dubai.bits-pilani.ac.in (R.K.)

Table S1. XRD data for failure region analysis of all specimens at SS 316L HAZ.

2 theta (deg)	Height (cts)	FWHM (°2Th.)	d-Spacing (Å)	Rel. Int. (%)	Correlated Phase (s)	Miller Indices
			HT			
43.69	545.61	0.49	2.07	100	(Cr Ni), FeNi, FeC	A 1 1 1
51.03	45.37	0.57	1.79	8.26	(Cr Ni), FeNi, $Cr_{23}C_6$	A 2 0 0
74.66	10.38	0.65	1.27	1.89	(Cr Ni), FeNi	A 2 0 2
90.77	32.69	0.82	1.08	5.95	(Cr Ni), FeNi	A 3 1 1
96.12	21.79	0.82	1.04	3.97	(Cr Ni)	M 2 2 2
			CT			
44.31	388.85	0.49	2.04	100	(Cr Ni), FeNi, Fe_3C	A 1 1 1
51.59	40.39	0.49	1.77	65.82	$Cr_{23}C_6$, Cr Ni, FeNi	A 2 0 0
75.69	9.24	0.65	1.26	15.95	FeNi, Cr Ni	A 2 0 2
91.93	29.1	0.65	1.07	20.68	$Cr_{23}C_6$, (Cr Ni),FeNi	A 3 1 1
97.27	19.4	0.67	1.03	4.43	(Cr Ni)	M 2 2 2
			UT			
43.51	1000.00	0.476	2.12	100	(Cr Ni), FeNi	A 1 1 1
50.7	104.3	0.714	1.83	8.45	$Cr_{23}C_6$, (Cr Ni), FeNi	A 2 0 0
74.76	40.2	0.782	1.3	1.93	FeNi, Cr Ni)	A 2 0 2
90.89	65.2	0.85	1.1	6.09	$Cr_{23}C_6$, Cr Ni, FeNi	A 3 1 1
96.14	45.7	0.986	1.06	4.06	(Cr Ni)	M 2 2 2
